# A patient with a purple, edematous great toe

**DOI:** 10.1016/j.jdcr.2025.04.044

**Published:** 2025-06-16

**Authors:** Hannah Bulosan, Yadira Castaneda Nunez, Bicong Wu, Evan George, Anna L. Cogen

**Affiliations:** a Department of Dermatology, University of Washington, Seattle, Washington; b Department of Medical Oncology, Fred Hutch Cancer Center, Seattle, Washington; c Department of Pathology, University of Washington, Seattle, Washington

**Keywords:** granulomas, sarcoidosis

## History

**Fig 1:**
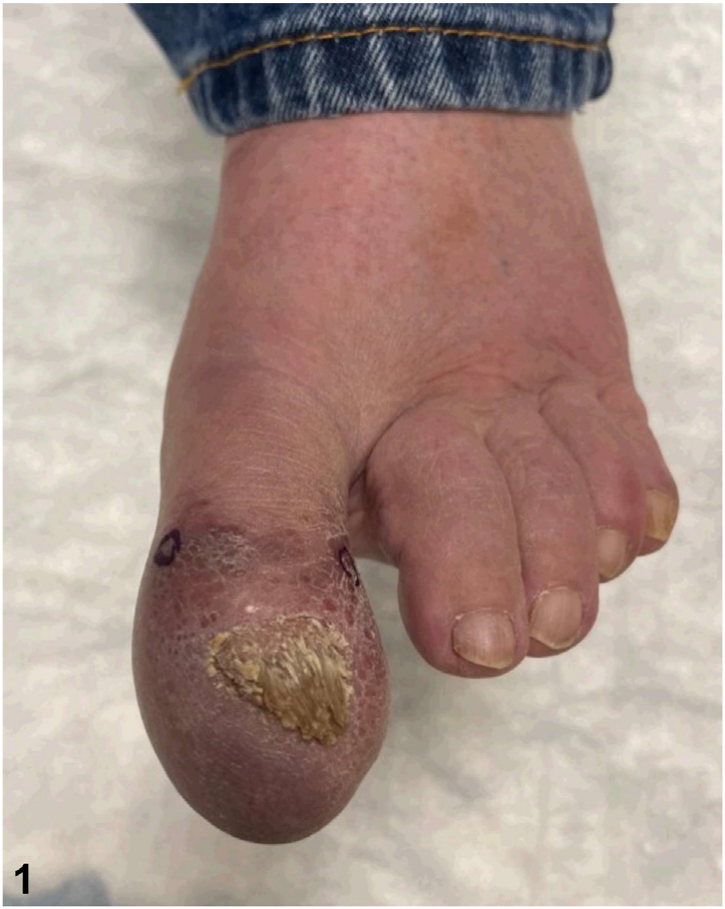

**Fig 2:**
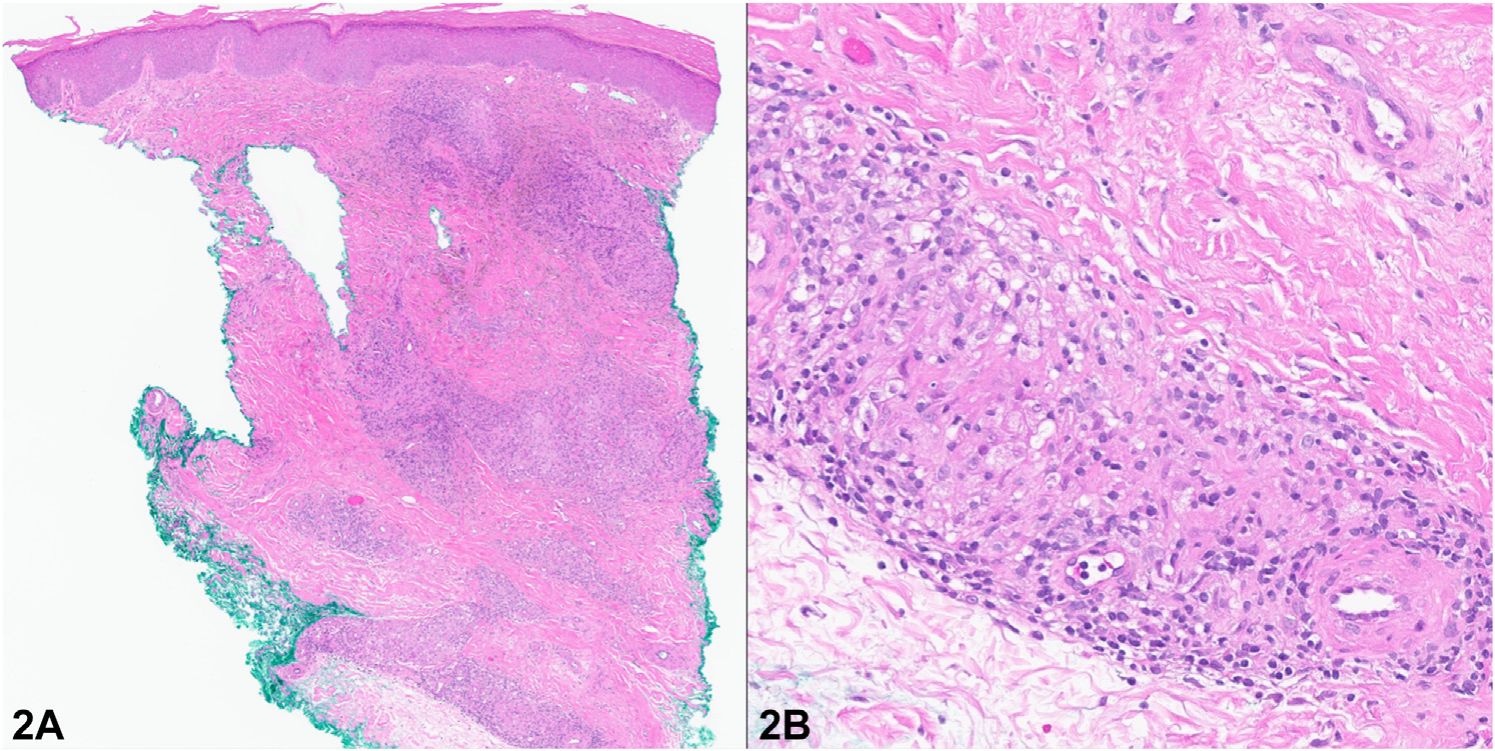

A 69-year-old male with Waldenström macroglobulinemia and pulmonary sarcoidosis presented with a 10-year history of intermittent swelling and discoloration of the left great toe, which recently worsened. Examination of the toe revealed diffuse edema with violaceous discoloration, and a thickened, dystrophic nail plate (Fig 1). Radiography of the toe revealed soft tissue swelling without bone involvement. C-reactive protein and erythrocyte sedimentation rate were within normal limits. Punch biopsies of the toe were performed for histopathology and tissue cultures (Fig 2). Special stains, including Gomori methenamine silver, acid-fast bacilli, and human herpesvirus-8, were negative, and comprehensive tissue cultures yielded no growth.

**Question 1: What is the most likely diagnosis based on the clinical presentation and histologic findings?**

A. Cryoglobulinemia
B. Atypical mycobacterial infection
C. Gout
D. Sarcoidosis
E. Kaposi sarcoma

**Answers:**

A. Cryoglobulinemia – Incorrect. Waldenström macroglobulinemia can lead to type I cryoglobulinemia with cutaneous manifestations, including retiform purpura on areas such as acral sites, ulcers, infarction, and hemorrhagic crusts. However, histopathologic findings typically show vasculitis or noninflammatory hyaline thrombosis. These features are inconsistent with this patient’s biopsy.
B. Atypical mycobacterial infection – Incorrect. Infection is a consideration given his history of Waldenström macroglobulinemia. However, the biopsy lacks necrotizing granulomas or organisms, which would be expected in an infectious process.
C. Gout – Incorrect. While the great toe is a common location for gout, it typically presents as acute, painful monoarthritis with monosodium urate crystal deposition. Histology would show amorphous crystalline deposits with a foreign-body granulomatous reaction, which is inconsistent with this patient's chronic history and biopsy findings.
D. Sarcoidosis – Correct. While the clinical presentation of a violaceous, swollen toe with nail dystrophy is not specific for sarcoidosis, the presence of noncaseating granulomas on histopathology, along with the patient’s known history of systemic sarcoidosis, strongly supports this diagnosis. Specifically, this presentation suggests sarcoid dactylitis, a rare manifestation occurring in approximately 0.2% of all sarcoidosis cases, typically involving the digits with swelling, pain, and discoloration.^1^^,^^2^
E. Kaposi sarcoma – Incorrect. While Kaposi sarcoma often presents as violaceous plaques or nodules on the distal extremities, particularly in immunocompromised individuals, histopathology would typically reveal spindle cell and vascular proliferation, features not observed in this case.

**Question 2: Which of the following is considered the first-line treatment for acute sarcoid dactylitis?**

A. Tumor necrosis factor-alpha (TNF-α) inhibitors
B. Methotrexate
C. Systemic corticosteroids
D. Calcium channel blockers
E. Sulfasalazine

**Answers:**

A. Tumor necrosis factor-alpha (TNF-α) inhibitors – Incorrect. TNF- α inhibitors may be used for long-term management but are not considered first-line therapy. Although no clinical trials have established an optimal therapy, both infliximab and adalimumab have been reported in the treatment of sarcoidosis, including cases with musculoskeletal involvement.^2^
B. Methotrexate – Incorrect. This agent may be used in refractory or chronic cases but is not the initial treatment choice.^3^
C. Systemic corticosteroids – Correct. Systemic corticosteroids are considered the first-line treatment for acute sarcoid dactylitis. Treatment often involves oral corticosteroids in the range of 15 to 20 mg per day, with dosing adjusted based on clinical response.^3^ Higher doses have been used in cases of more active or severe disease.^2^
D. Calcium channel blockers – Incorrect. These are used for conditions like pernio and Raynaud’s phenomenon but have no established role in sarcoid dactylitis treatment.
E. Sulfasalazine – Incorrect. While sulfasalazine is used in the management of inflammatory arthritis, it has no recognized role in the treatment of sarcoid dactylitis.

**Question 3: Which of the following is most likely to be seen on imaging of sarcoid dactylitis?**

A. Osteophyte formation and joint space narrowing
B. Soft tissue fusiform swelling with bone erosions
C. Juxta-articular bone proliferation with “pencil-in-cup” deformity
D. Periarticular osteopenia with marginal erosions and joint space narrowing
E. Soft tissue calcification

**Answers:**

A. Osteophyte formation and joint space narrowing – Incorrect. These findings are typically seen in osteoarthritis.
B. Soft tissue fusiform swelling with bone erosions – Correct. Sarcoid dactylitis typically involves both soft tissue and bone of the fingers, most commonly presenting as bilateral fusiform or sausage-shaped swelling.^1^ Granulomatous inflammation within the soft tissues adjacent to the bone can lead to destruction, resulting in characteristic radiographic findings such as punched-out or cystic bone lesions.^4^ Although our case did not show bone involvement on x-ray, the diagnosis of sarcoidosis was confirmed histologically through biopsy. While bone involvement is a recognized feature of sarcoid dactylitis, its exact prevalence remains unclear, and cases without radiographic evidence have been reported.^5^
C. Juxta-articular bone proliferation with “pencil-in-cup” deformity – Incorrect. These are classic findings seen in psoriatic arthritis.
D. Periarticular osteopenia with marginal erosions and joint space narrowing – Incorrect. These findings are characteristic of rheumatoid arthritis, which commonly involves proximal interphalangeal and metacarpophalangeal joints.
E. Soft tissue calcification – Incorrect. Soft tissue calcification may be present in a wide range of pathologies such as pseudogout, osteosarcoma, or myositis ossificans, but is not typical of sarcoid dactylitis.

## Conflicts of interest

None disclosed.

## References

[1] Weaver J., Morris E., Raimer S.S., Colome-Grimmer M.I. Drumstick dactylitis: an unusual presentation of sarcoid. Internet J Dermatol. 2003;2(2). https://ispub.com/IJD/2/2/3676.

[2] Alawneh D., Al-Shyoukh A., Edrees A. (2020). TNF inhibitor treating osseous sarcoidosis and dactylitis: case and literature review. Clin Rheumatol.

[3] Nessrine A., Zahra A.F., Taoufik H. (2014). Musculoskeletal involvement in sarcoidosis. J Bras Pneumol.

[4] Bechman K., Christidis D., Walsh S., Birring S.S., Galloway J. (2018). A review of the musculoskeletal manifestations of sarcoidosis. Rheumatology.

[5] Curco N., Pagerols X., Vives P. (1995). Subcutaneous sarcoidosis with dactylitis. Clin Exp Dermatol.

